# The first principle calculation of improving p-type characteristics of B_*x*_Al_1-*x*_N

**DOI:** 10.1038/s41598-021-92260-6

**Published:** 2021-06-16

**Authors:** Zhengqian Lu, Fang Wang, Yuhuai Liu

**Affiliations:** 1 National Center for International Joint Research of Electronic Materials and Systems, Zhengzhou University, Zhengzhou, Henan China; 2 International Joint-Laboratory of Electronic Materials and Systems of Henan Province, Zhengzhou University, Zhengzhou, Henan China; 3 Department of Information and Communication Engineering, School of Information Engineering, Zhengzhou University, Zhengzhou, Henan China; 4 Zhengzhou Way Electronics Co. Ltd., Zhengzhou, Henan China

**Keywords:** Materials science, Theory and computation, Electronic structure

## Abstract

AlN is one of the third-generation semiconductor materials with wide application prospects due to its 6.2 eV band gap. In the application of semiconductor deep ultraviolet lasers, progress is slow due to the difficulty in obtaining p-type AlN with good performance. In this paper, the commonly used way of Mg directly as AlN dopant is abandoned, the inhibition effect of the B component on self-compensation of AlN crystal was studied. The improvement of self-compensation performance of AlN crystal by B component is studied by first principles calculation. The results show that the addition of B component can increase the hole concentration of AlN, which is conducive to the formation of p-type AlN.

## Introduction

AlN has the widest band gap (6.2 eV) among the Group-III nitride semiconductors. Therefore, AlN plays a significant role in the development of solid-state ultraviolet light sources for light-emitting diodes and laser diodes. For such applications, both conductive n-and p-type AlN materials are required. The n-type AlN can be achieved by using traditional doping techniques that exchange atoms with impurities of additional electrons, such as Si doping^1–3^, but the p-type AlN remains a major challenge.

At present, the following factors restrict the conductivity of p-type AlN: limited solubility in related receptors, high activation energy of acceptors, and compensation for impurities and natural donor defects^4^. In general, the p-type doping of III-nitride semiconductors is achieved by substituting dopants for the primary atom, which have fewer valence electrons than the primary atom^5^. For example, p-type GaN can be reproduced by MOCVD, Mg doping and annealing^6^. The activation energy of Mg receptor in GaN is about 160 meV, while that in AlN is about 500 meV^7–10^. Recent reports on the preparation of p-type AlN by Mg doping show that these empty points in the impurity band have a very high activation energy of about 600 meV and a low mobility^11^, which makes Mg-doped AlN ineffective in practical applications^12,13^. The results show that the void concentration at room temperature is about 10^10^ cm^−3^ in Mg-doped AlN.

To obtain p-type AlN with better conductivity, the self-compensation effect of AlN needs to be solved first. It is assumed that the main reason for the self-compensation effect of AlN is that the electronegativity of the Al and N atoms are different. At the process of electron hybridization, the electrons of the N atom cannot be well bound, which makes the electrons of the N atom relatively free, thus causing a serious self-compensation effect. By first-principles calculation, we try to prove that substituting Al atom with B atom (electronegativity is higher than Al atom) can increase the hole concentration, restrain the self-compensation effect.

## Methodology

This paper uses the first principles to calculate the band bowing of BAlN with different composition of B. In order to obtain data of low concentration B composition, the supercell structure is too large to be calculated. Therefore, using virtual crystal as the calculation model, very low B composition can be obtained without changing the periodicity of the crystal. Because this paper involves the calculation of low component b-band structure, the DFT calculation system is too large to distinguish the energy band, and the special B atom position, especially when there are more than two B atoms, the different position relationship will affect the energy band structure, so the virtual crystal is used to average the components to avoid the above situation. With this method, the lattice constants and band structure of BAlN with different B composition are calculated. Among them, the valence electrons of Al atom are $3d^{2} 3p^{1}$, N atom is $2s^{2} 2p^{3}$, and B atom is $2s^{2} 2p^{1}$.

**Figure 1:**
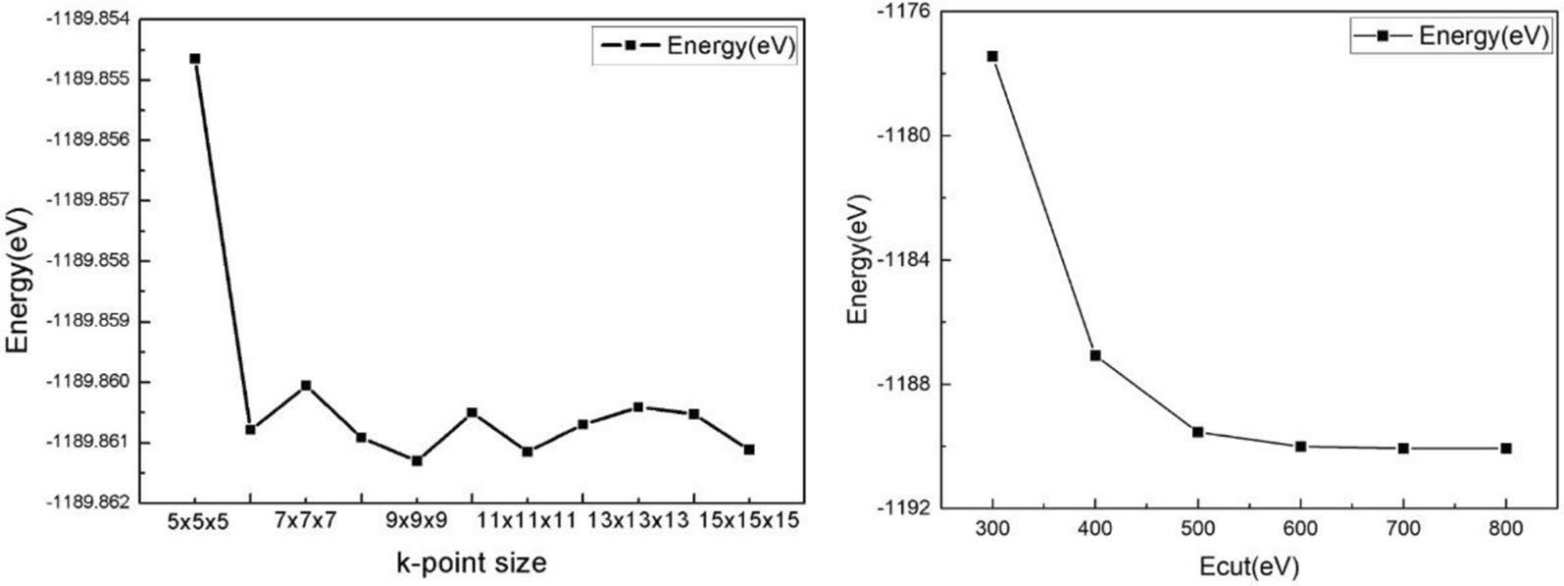
K point and cut-off energy test.

From Fig. 1, it is possible that the energy calculation of the cell will vary with the selection of different K-point sizes and the Cut-off energy sizes. For a single cell, the k-point oscillation near a certain value is a normal. If the k-point is too close, there will be spatial overlap. And the lowest energy state is obtained when the Cut-off energy is 500 eV at 9 × 9 × 9 K-point size, but the K-point oscillation beginning at 6 × 6 × 6. The Cut-off energy tends to converge at 600 eV while maintaining 6 × 6 × 6 K-point size. Although it is not in the lowest energy state, but the increase of Cut-off energy will increase the computational load of the computer, so using 600 eV as the Cut-off energy can guarantee both low energy state and not giving too much load to the computer.

Different lattice optimization results for the original cell can be obtained by using different exchange–correlation potentials, as shown in Table 1. It can be seen that different exchange–correlation potentials can result in different optimized lattice constants, among which the GGA is the closest to the experimental values, so this algorithm is used in the crystal optimization process. After determining the optimization algorithm, comparing the band characteristics calculated for different exchange–correlation potentials yields results such as Table 2.

**Table 1:** Lattice optimization results.

	GGA	LDA	HSE03	HSE06	Exp
**AlN**					
a(Å)	3.079	3.066	3.014	3.008	3.11^a^
c(Å)	4.938	4.917	4.834	4.823	4.98^a^
**BN**					
a(Å)	2.565	2.531	2.512	2.507	(2.55)^b^
c(Å)	4.245	4.189	4.157	4.148	(4.20)^b^

^a^Ref^14,15^.

^b^Ref^16^.

() Means no experimental data.

**Table 2:** calculation results of energy gap.

	LDA	PBE	RPBE	PW91	BLYP	HSE06	Exp
**AlN**							
Eg(eV)	4.18	4.134	4.218	4.183	4.525	6.01	6.2^a^
**BN**							
Eg(eV)	4.941	5.246	5.385	5.291	5.678	6.398	(6.86)^b^

^a^Ref^15,17^.

^b^Ref^18^.

() Means no experimental data.

From Table 2, it can be seen that the optimal band gap can be obtained by the exchange–correlation potential of HSE06, but the algorithm cannot be used for the calculation of virtual crystals, HSE06 is nonlocal exchange function can only be used for insulators using the all-bands minimizer (not density mixing) with the Energy, Geometry Optimization. And virtual crystals is an atom mixing two type element. So only the BLYP exchange–correlation potential closest to the experimental value can be selected as the calculation method of the band.

## Results and discussion

AlN and BN are both crystals with p63mc space group, but the BN of wurtzite is very unstable and difficult to obtain in the experiment, so the first principle is particularly important for its research. The two crystals have the same structure and can be studied as alloys. Because the electronegativity of B atom is 2.051, which is much larger than that of Al atom, if B atom is used to replace Al atom in AlN, a positive electric center will be formed, which is beneficial to improve the self-compensation effect of AlN and make p-type doping of AlN possible.

**Figure 2:**
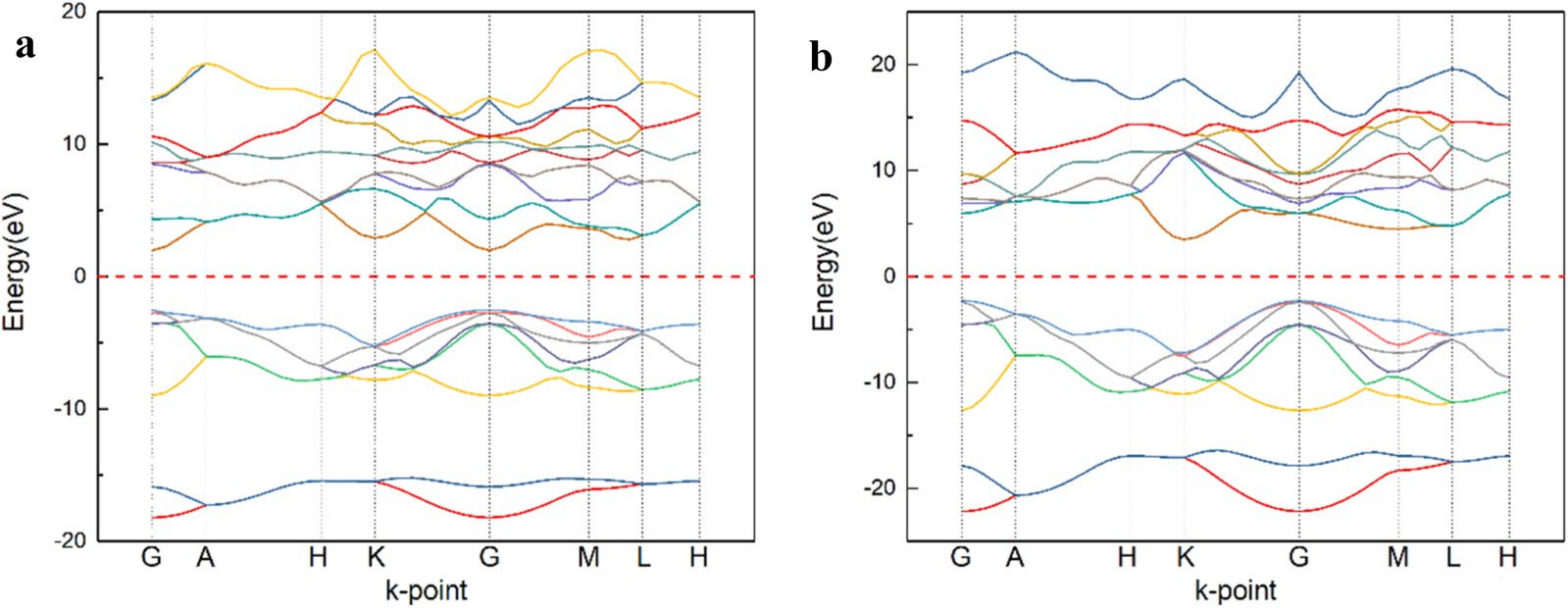
Band structure of (**a**) AlN, (**b**) BN crystal.

Firstly, the crystal band structures of AlN and BN with wurtzite structure are calculated. In Fig. 2, the bands of different colors represent the bands of different electrons. And in the DFT calculations can only consider the electronic properties at 0 K, and defines Fermi energy level as the highest occupying state of electrons. The Fermi-level should be very close to the VBM under this condition. The change of carrier can't be compared directly, so in this paper, the Fermi level is the position where the electron and hole appear with equal probability. The alloy formed by doping B belongs to high concentration impurity and degenerate semiconductor. The electron hole obeys Fermi–Dirac distribution. Combining with the state of density, the energy width of the equal charge number at the top of the valence band and the bottom of the guide band is selected as the reference object to calculate the Fermi energy level. And the Fermi level in Fig. 2 is near the center. It can be seen that BN crystal is an indirect band gap, and at the G-point the Fermi energy level is closer to the top of the valence band. AlN crystal has a direct band gap, and the Fermi energy level is in the middle of the conduction band and valence band, and is slightly close to the conduction band, showing n-type characteristics. Hence, with the increase of B composition in ${\text{B}}_{x} {\text{Al}}_{{1 - x}} {\text{N}}$ alloys, the transition from direct band gap to indirect band gap will be formed. According to reference^19^, B composition in BAlN compound is still a direct band gap semiconductor when it is lower than 28%. Thus, it is possible that the increase of B component will raise the conduction band at G-point of AlN crystal and increase the hole concentration.

**Figure 3:**
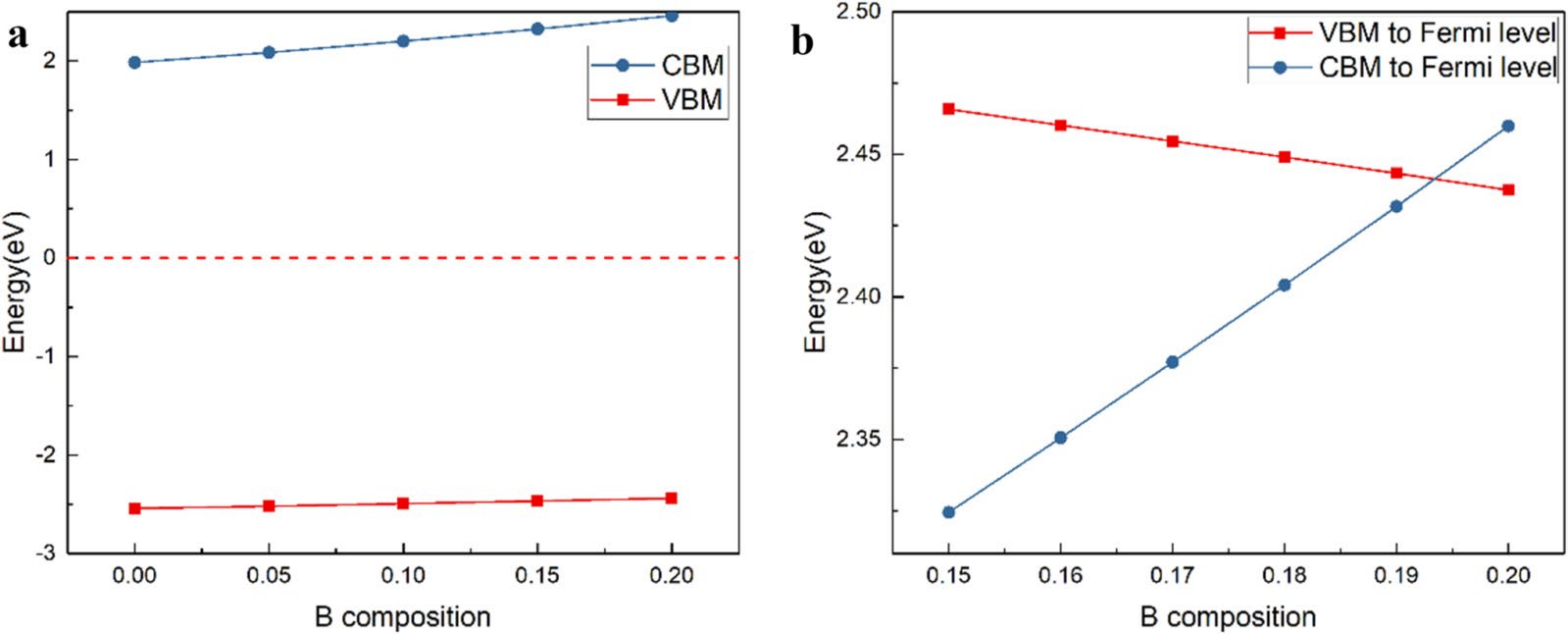
Energy band of different composition.

Figure 3 can be obtained by calculating the position relationship between the top of the valence band and the bottom of conduction band when the value of x is from 0 to 0.2 with the interval of 0.05 for B composition by the method of virtual crystal. In reference^20^, it was reported that 14.4% of wurtzite structure BAlN was prepared by high temperature and small B / III method, so this paper did not consider the cubic phase transition of high B component. In Fig. 3a, it shows the position relationship between the top of the conduction band, the bottom of valence band and Fermi level for different B composition. In Fig. 3b, the solid line represents the energy difference between the bottom of the conduction band and the Fermi level; the dotted line represents the energy difference between the Fermi level and the top of the valence band. It can be seen from the figure that when composition of B is below 0.15, the difference between the valence band top and Fermi level of BAlN is still greater than that between the conduction band top and Fermi level, with the increase of B component, the difference between them has a decreasing trend. When the composition is increased to 0.2, the difference between the valence band top and Fermi level becomes smaller than that between the conduction band top and Fermi level, and the concentration of hole is higher than that of electron in the compound. It can be seen from the b-diagram that the B component is near about 19.5%, the VBM is equal to CBM.

**Figure 4:**
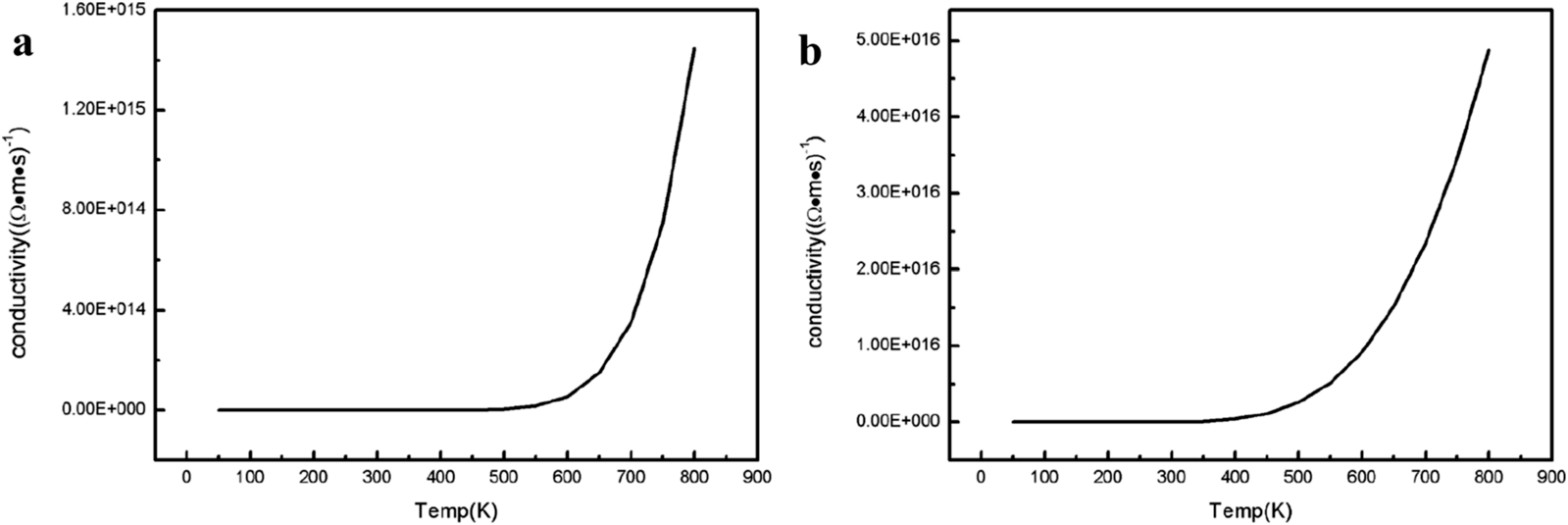
conductivity (**a**) AlN, (**b**) B_0.2_Al_0.8_ N.

Then the conductivity of AlN crystal and 20% BAlN alloy is calculated by BoltzTrap module^21^. It can be seen from Fig. 4 that the conductivity curve of 20% BAlN alloy rises faster than that of AlN. At 300 K, the conductivity of AlN is 2.69 × 10^8^ (Ω m s)^-1^, while that of BAlN is 2.12 × 10^12^ (Ω m s)^-1^. It is reasonable to believe that the addition of component B may improve the conductivity of AlN.

When there are acceptor (A), donor (D), Al vacancy and N vacancy in AlN crystal, the ionization equation is as follows:

$$ A^{{ - i}}  \leftrightarrow A^{{ - i - 1}}  + h $$

$$ D^{{ + i}}  \leftrightarrow D^{{ + i + 1}}  + e $$

$$ V_{{Al}}^{{ - i}}  + e \leftrightarrow V_{{Al}}^{{ - i - 1}} $$

$$ V_{N}^{{ + i}}  + h \leftrightarrow V_{N}^{{ + i + 1}} $$

$$ e + h \leftrightarrow o $$

where $A^{{ - i}}$, $D^{{ + i}}$, $V_{{Al}}^{{ - i}}$, $V_{N}^{{ + i}}$, is acceptor (A), donor (D), Al vacancy and N vacancy of i valence state, e, h, o is electron, hole and recombination. According to the Fermi statistical mechanics equation:

$$ A^{{ - i}} /A^{{ - i - 1}}  = g_{A}^{{i + 1}} /g_{A}^{i} exp\left( {E_{A}^{{i + 1}}  - E_{F} } \right)/kT $$

$$ D^{{ + i}} /D^{{ + i + 1}}  = g_{D}^{{i + 1}} /g_{D}^{i} exp\left( {E_{F}  - E_{D}^{{i + 1}} } \right)/kT $$

$$ V_{{Al}}^{{ - i}} /V_{{Al}}^{{ - i - 1}}  = g_{{Al}}^{{ - i}} /g_{{Al}}^{{ - i - 1}} exp\left( {E_{{VAl}}^{{ - i - 1}}  - E_{F} } \right)/kT $$

$$ V_{N}^{{ + i}} /V_{N}^{{ + i + 1}}  = g_{{VN}}^{{ + i}} /g_{{VN}}^{{ + i + 1}} exp\left( {E_{F}  - E_{{VN}}^{{i + 1}} } \right)/kT $$

$$ {\text{n}} = N_{c} {\text{exp}}\left( {E_{F}  - E_{c} } \right)/kT $$

$$ {\text{p}} = N_{v} {\text{exp}}\left( {E_{v}  - E_{F} } \right)/kT $$

 <  > denotes concentration, A is acceptor, D is donor, p and n are hole and electron concentration, $g^{i}$ is the degradation degree of i valence defect energy level $E^{{i + 1}}$ ionization energy from i valence to i + 1 valence,${\text{~}}N_{c}$ and $N_{v}$ is the density of states at the bottom of conduction band and the top of valence band. When there are J kinds of donor impurities and K kinds of acceptor impurities in the crystal, according to the electric neutral condition, it can be concluded that:

$$ \mathop \sum \limits_{{i \ge 1}} i\left\{ {\mathop \sum \limits_{{j = 1}}^{J} \left[ {D^{{ + i}} } \right]_{j}  + \left[ {V_{N}^{{ + i}} } \right]} \right\} + p = \mathop \sum \limits_{{i \ge 1}} i\left\{ {\mathop \sum \limits_{{k = 1}}^{K} \left[ {A^{{ - i}} } \right]_{k}  + \left[ {V_{{Al}}^{{ - i}} } \right]} \right\} + n $$

Because the electronegativity of B element is greater than that of Al element, doping will form acceptor level. When only ionized acceptor and N vacancy are considered, we can get $A^{ - }  = V_{N}^{{ + 1}}  + 2V_{N}^{{ + 2}}  + 3V_{N}^{{ + 3}}  + p$. $p/A^{ - }$ denotes the degree of self-compensation. The larger this term is, the lower the degree of self-compensation is. As an equal charge doping, B atom forms a negative center but does not change the valence state due to the increase of negative charge. Therefore, it is assumed that $A^{ - }$ does not increase significantly, but the hole concentration is increased. It is speculated that B doping can reduce the self-compensation effect of AlN.

**Figure 5:**
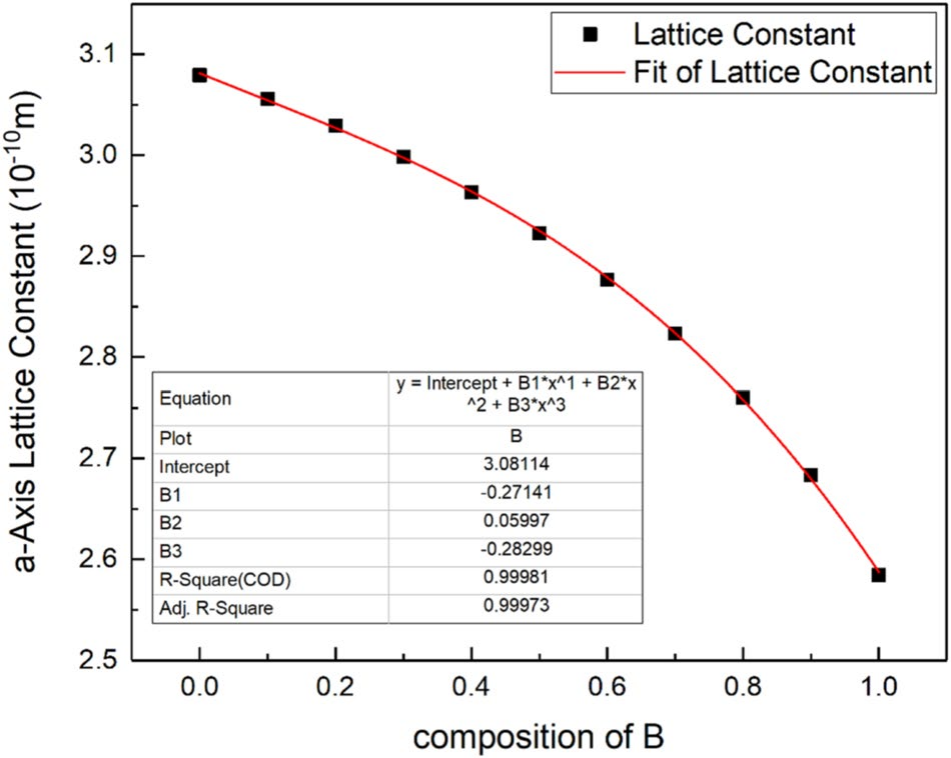
a-Axis lattice constant changing with B composition.

Through the calculation of lattice optimization for different B composition virtual crystals, the change rule of lattice constant in a-Axis can be obtained, as shown in Fig. 5. The formula for the change of lattice constant of a-Axis with the increase of B composition can be obtained by fitting:

$$ a = 3.081 - 0.271x + 0.06x^{2}  - 0.283x^{3} $$

When the B composition is 20%, the lattice mismatch of AlN crystal and BAlN crystal is only 1.6%.

## Conclusions

The electronegativity difference between Al atom and N atom may be too large in AlN crystal. During the formation of the crystal, the hybrid electrons of Al atom and N atom are not well bound in the hybrid process, showing certain free-electron characteristics. Therefore, there is a strong self-compensation characteristic in the process of Mg doping. Subsequently, using B with higher electronegativity as a dopant can not only replace Al atom to form a positive center, but also increase the binding ability to electrons. When the B composition reaches 19.5%, ${\text{B}}_{x} {\text{Al}}_{{1 - x}} {\text{N}}$ the VBM is equal to CBM. At the same time, it has been reported that when the B composition is less than 28%, the band gap of ${\text{B}}_{x} {\text{Al}}_{{1 - x}} {\text{N}}$ is direct. When the a-axis lattice constant of the compound is calculated, it can be found that the lattice mismatch with AlN crystal is only 1.6% at 19.5% B composition. Thus, using boron as doping material can effectively improve the hole concentration of AlN crystal. This is very effective for the preparation of p-AlN crystal.
